# Complete Genome Sequence of *Bacillus vallismortis* NBIF-001, a Novel Strain from Shangri-La, China, That Has High Activity against *Fusarium oxysporum*

**DOI:** 10.1128/genomeA.01305-17

**Published:** 2017-11-30

**Authors:** Xiaoyan Liu, Yong Min, Daye Huang, Ronghua Zhou, Wei Fang, Cuijun Liu, Ben Rao, Guangyang Zhang, Kaimei Wang, Ziwen Yang

**Affiliations:** a National Biopesticide Engineering Technology Research Center, Hubei Biopesticide Engineering Research Center, Hubei Academy of Agricultural Sciences, Biopesticide Branch of Hubei Innovation Centre of Agricultural Science and Technology, Wuhan, China; b Hubei Academy of Agricultural Sciences, Wuhan, China

## Abstract

*Bacillus vallismortis* NBIF-001, a Gram-positive bacterium, was isolated from soil in Shangri-La, China. Here, we provide the complete genome sequence of this bacterium, which has a 3,929,787-bp-long genome, including 4,030 protein-coding genes and 195 RNA genes. This strain possesses a number of genes encoding virulence factors of pathogens.

## GENOME ANNOUNCEMENT

*Bacillus vallismortis* is a bacterial species in the genus *Bacillus* with a high affinity for *B. subtilis* (1). In its growth process, *B. vallismortis* can produce numerous antimicrobial bioactive metabolites, such as thermostable alkalophilic cellulase and bacillomycin D (2, 3). These antibiotic compounds may provide alternative resources for the biocontrol of plant diseases (4, 5). Strain NBIF-001 belongs to the species *B. vallismortis*. The type strain of the species produces many bioactive metabolites showing specific activity against *Fusarium oxysporum* f. sp.* niveum*, which causes watermelon wilt disease, the most destructive disease to melon crops worldwide (6, 7).

Strain NBIF-001 was isolated from soil in Shangri-La, China, in October 2010. Its 16S rRNA sequence has 99.0% similarity to that of *B. vallismortis*. The genome sequencing of *B. vallismortis* NBIF-001 was performed with a strategy involving the Paciﬁc Biosciences (PacBio) RS II sequencing platform and Solexa paired-end sequencing technology. Two libraries containing 10-kb and 400-bp inserts were constructed. Sequencing with an Illumina Solexa IIx genome analyzer (Wuhan Yanxing Biotechnology Co., Ltd., Wuhan, China) was performed using a paired-end strategy of 500-bp reads to produce 571 Mb and 622 Mb of ﬁltered sequences, representing 192-fold coverage. Reads were then *de novo* assembled using the Hierarchical Genome Assembly Process (HGAP) version 3.0 to generate one contig without gaps (8).

The assembled NBIF-001 genome revealed a single circular chromosome with a size of 3,929,787 bp and an overall G+C content of 46.5%. A total of 4,030 protein-coding genes were predicted in the genome, and 86 tRNAs, 27 rRNAs, and 82 other noncoding RNAs were found in the chromosome. Genome annotation was performed with the NCBI Prokaryotic Genome Automatic Annotation Pipeline (PGAAP), and the NCBI nonredundant (NR), KEGG (9), and Clusters of Orthologous Groups (COG) (10) databases were employed for BLASTp identiﬁcation (11). Of the 4,030 genes, 3,285 were classiﬁed into 21 functional categories based on COGs of proteins (12). The genome contained 11 virulence genes relative to the resistance of pathogens, *clpC*, *clpE*, *galE*, *lplA1*, *acpXL*, *bslA*, *yuaB*, *clpP*, *cps4I*, *capC*, and *ureB*. Moreover, there are 64 antibiotic resistance genes, including 39 genes on the antibiotic resistance, 22 genes on the antibiotic target, and 3 genes on the antibiotic biosynthesis; 11 gene clusters were found to direct the synthesis of inhibition of plant pathogen factors, including surfactin, butirosin, terpene, lantipeptide, macrolactin, bacillaene, fengycin, iturin, difficidin, bacillibactin, and bacilysin.

### Accession number(s).

The complete genome sequence of *B. vallismortis* NBIF-001 has been deposited in GenBank under the accession number CP020893. The strain is available from the China Center for Type Culture Collection (CCTCC) in Wuhan under the accession number CCTCC M 2015087.
